# The Explanation of Photopic Luminous Efficiency Curve by Using Both of the Cones’ Optical Fiber Coupling Effects and the Absorption of L Cones

**DOI:** 10.3390/s23031523

**Published:** 2023-01-30

**Authors:** Anhui Liang, Kexin Yu, Xiaolin Min, Jing Li, Jianyu Li, Xiaoling Zuo, Youli Yao

**Affiliations:** College of Electronic Information Engineering, Shandong University of Science and Technology, Qingdao 266590, China

**Keywords:** vision, color vision, cone, optical fiber, retina

## Abstract

In this paper, we build four-part cone models to explore the coupling effect of seven cone fiber couplers. Moreover, this is the first study of the coupling effect of four layers of biological couplers in animals and other biological lives. We simulate the four layers cone couplers by using the beam propagation method, and we assume the input beam is located at the outer fiber of the central cone. Our simulation results showed that there are two wavelength regions (short and long wavelength regions) with the strongest coupling, where the most power of input optical powers of the central cones will transfer to the six surrounding cones after transmitting through the four layers of cone couplers. However, within a wavelength region of ±75 nm near to the peak wavelengths, located in the yellow–green wavelength range, the splitting ratios at the output of the outer segment of the central cone are always greater than the sum of the splitting ratios of the six surrounding cones. These cone couplers may play an important role in color preprocessing (e.g., doing opponent color processing partially). The cone fiber coupler effect and light absorption of cones are considered separately in our models. By taking account of both the cone fiber coupling effect and absorption of outer segment of L cone, we find the multiplication of the relative optical power of cone couplers, the spectral sensitivity data of the L cone, and a normalized coefficient that matches with the photopic luminous efficiency of the human eye well. This is the attempt to use both the cone fiber coupling effect and the absorption of L cones to explain the photopic luminous efficiency. The splitting ratios of the central cones are greater than 80% at peak wavelengths located in the yellow-green wavelength range, and this can help to explain why the human eye is more sensitive to green light.

## 1. Introduction

In 1961, Enoch first demonstrated that the cone cells of the human eye were optical waveguides [1]. Harte then found that photoreceptors had the properties of semiconductors [2]. Later, the optical fiber properties of human eye photoreceptors were studied. In 1998, Liang first proposed that not only the inner segments of the three types of cone cells in the fovea of the human eye, but also the outer segments of the L and M cones have the properties of single-mode fibers [3]. Liang was the first to study the optical fiber coupling effects of human and vertebrate cones [4,5,6,7,8,9,10]. In 2013, Liang studied the coupling of the outer segment of a model consisting of two cones and later studied two- and seven-cone fiber coupler models of the ellipsoid, myoid, and outer fiber. We called the strong optical coupling region as the color preprocessing stage [7]. By adding the color preprocessing stage before the traditional three stages (stage theory), Liang first propose the four-stage theory to unify the tricolor theory and opponent color theory of color vision [7]. Moreover, Liang first proposed that the inverted retinal structure of humans and vertebrates was a bio-optical artificial intelligence (AI), consisting of four layers of fiber-optic couplers among cones and saturable absorbers in the outer segment. This AI structure enables the human eye to reduce energy consumption, heat generation, and latency. The Stiles–Crawford I effect describes that photoreceptors of the human eye are angularly selective to incident light [11]. Liang et al. found that the ellipsoid, with many mitochondria, of a human central foveal cone acts as a spot size converter to reduce coupling loss between myoid and outer segment [6]. In 2022, Ball et al. studied the microlens effect of a squirrel cone’s ellipsoid, which is with larger diameter than that of a human foveal cone’ ellipsoid, and its roles in the Stiles-Crawford I effect [11]. The bio-optical waveguides and couplers have also been studied in other fields as well. For example, Xin et al. first used *Escherichia coli* to make biological optical fiber couplers consisting of bacteria [12,13,14]. There are also biological optical waveguides in the leaves and stems of plants to receive sunlight and transmit sunlight to their roots [15].

There are two main types of photoreceptors in the human eye: cones and rods. Unlike the rods, which have only one type, the cones are classified into L, M, and S cones due to their sensitivity to different wavelengths of light. According to the trichromatic theory, three types of cones stimulated by different wavelengths of light would produce psychologically different color perceptions [16,17,18]. The L, M, and S cones are sensitive to red, green, and blue, with the most sensitive wavelength peaks at approximately 564, 532, and 438 nm, respectively [18,19,20]. In the foveal, there are only cones but no rods. Photopic vision is the vision of the eye under a bright environment (luminance above 3 cd/m^2^), where the cone cells play a major role. Scotopic vision relates to a dim environment (luminance below 10^−3^ cd/m^2^), where the rod cells play a major role. When the conditions are somewhere in between, the cones and rods work together [21].

Photoreceptors act as optical waveguides [1,2,3,4,5,6,7,8,9,10,22,23,24]. Modal analysis of waveguide propagation has been used to study how light propagates through photoreceptors. The refractive index of photoreceptor cells is higher than that of the extracellular matrix [22,23,24,25,26,27,28]. Based on Liang’s theory, the inverted retinal structure of human and vertebrate eyes is a bio-optical AI, consisting of cone optical fiber couplers and saturable absorbers [10]. We called the strong cone optical coupling region as the color preprocessing stage, which may support opponent color processing. The wavelength dependence of optical coupling may reveal a novel mechanism for color sensation and color vision in the retina. This study may help to understand more color blindness and weakness as well. Liang first studied the cone fiber couplers consisting of seven three-part cones, which include outer fibers, myoids, and ellipsoids, but the coupling effect of the seven-cone optical coupler consisting of outer segments was not studied [10]. Although the coupling effect of the seven cone optical coupler consisting of outer segments is normally weaker than that consisted of the first front three-parts, it cannot be fully ignored, especially in the long wavelength region. In this paper, we build a four-part cone model to explore the coupling effect of seven cone fiber couplers, and this is the first to study the coupling effect of four layers of biological couplers in animals and other biological lives. We use the widely used commercial software Rsoft to perform the simulation, where the beam propagation method (BPM) is applied to study the fiber couplers [29]. By taking account of both the cone fiber coupling effect and absorption of outer segment of L cone, we find the multiplication of the relative optical power of cone couplers, the spectral sensitivity data of the L cone, and a normalized coefficient that matches with the photopic luminous efficiency of the human eye well. Indeed, this is the first attempt to use both the cone fiber coupling effect and the absorption of L cones to explain photopic luminous efficiency.

## 2. Materials and Methods

### 2.1. The Beam Propagation Method (BPM)

The beam propagation method (BPM) is a powerful tool for analyzing and studying optical waveguides. The BPM algorithm was proposed in the 1970s to deal with the laser transmission in the atmosphere and was then used to study the optical transmission in the waveguides [30]. The BPM algorithm has been improved by many scholars and widely used in the optical fiber industry. The BPM has become a very practical waveguide simulation algorithm tool, and its main feature is that it can deal with the waveguide and radiation modes in a unified manner.

The basic idea of BPM is to gradually calculate the field on each propagation cross-section under the premise of a given initial field. In the calculation, the waveguide section is divided into many grids represented by the difference equation. Then, the boundary conditions are added to solve this equation through numerical calculation, and the field distribution of the entire section is obtained. The smaller the step size of the waveguide section is, the higher the optical field accuracy between the section waveguides is.

The BPM is an efficient numerical method for solving the scalar Helmholtz equation in a three-dimensional space. It was used to simulate the propagation behavior of light in Müller cells as well [31].

### 2.2. Monitoring and Analysis of the Simulation Results

The BeamPROP simulation program in Rsoft software can calculate the light field propagating along waveguides. To analyze some physical quantities, such as optical power in a specific circuit area, the concept of monitors was introduced in BeamPROP. Although there are many types of monitors in BeamPROP, we use the Fiber Mode Power type. This type of monitor computes the overlap integral as a function of length between the simulated field and a test field which is an analytically computed fiber mode of the local position, and we use this result to determine the optical power the specified mode. In the simulation, we use a single cone as the propagation path and set up monitors in the outer segment of the cone for monitoring.

### 2.3. Parameters of the Models

**Figure 1:**
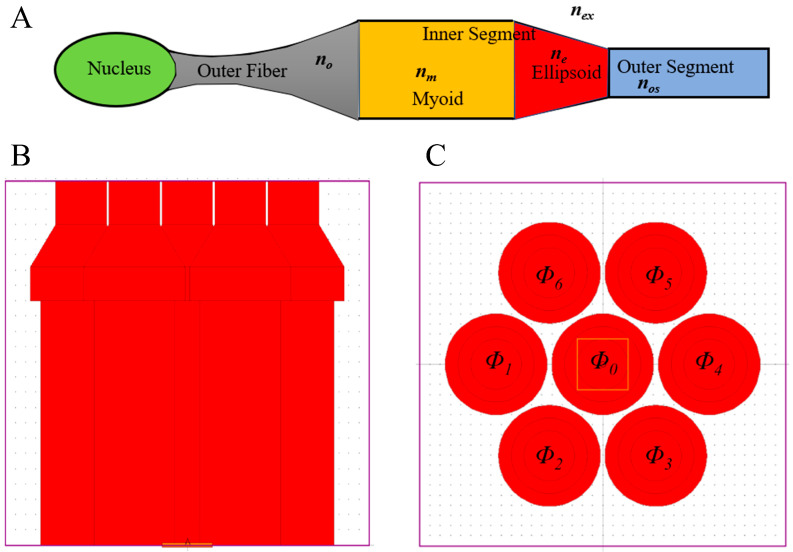
Structure and model diagram of cones. (**A**) Structural diagram of cone cells. (**B**) Four-parts model diagram established in Rsoft; the image corresponds to the outer segment, ellipsoid, myoid, and outer fiber from top to bottom; (**C**) is the top view of the (**B**). *Φ*_0_*~Φ*_6_ are the seven cones with the same parameters, which represent different positions. The orange square box in the figure represents the location of the light source, and the incident light enters the model from the bottom of the cone *Φ*_0_ at the center position and transmits to the outer segment. For the detailed parameters of the model, see the Materials and Methods.

Previously, the optical model of photoreceptor cells was divided into three-parts: the myoid, ellipsoid, and outer segments [24]. As shown in Figure 1A, Liang first proposed the five-part optical mode of vertebrate photoreceptors (including cones and rods) [4,5,6,7,8,9,10], where the five parts include nucleus, outer fiber, myoid, ellipsoid, and outer segments. The length of the photoreceptor nucleus is relatively short, and the nucleus acts as a lens to focus the incident light into the photoreceptor [32]. Therefore, the nucleus is not part of the strong later coupling region of fiber couplers, and the nucleus is not considered in the modeling of this paper. In this paper, we only study a four-part model, which includes outer fiber, myoid, ellipsoid, and outer segments [6,10]. However, the effect of the nucleus should be considered in future studies. The foveal of the retina is the most acute part of the vision, having only cone cells and no rod cells. Therefore, we focused on the optical properties of the cones. Since the size and length of the foveal cone cells differ with age, the average value of the size and length of adult cone cells are selected as the calculation basis.

It is generally believed that light absorption or photoelectric conversion occurs in the outer segment of the cone, while the light absorption in the outer fiber and inner segment is weak [7]. Therefore, in the model, the imaginary parts of the refractive index of outer fiber and inner segment are not considered. According to the literature, in the fovea of the human retina, the diameter and length of the outer fiber are *d_o_* = 1.6~2 μm and *l_o_* = 80~100 μm; the myoids are *d_m_* = 1.88~2 μm and *l_m_* = 11~16 μm; the outer segments are *d_os_* ≈ 1 μm, and the length of the ellipsoid is *l_e_* = 14~20 μm [7]. The distance between adjacent cones in the center of the human fovea is *δ_d_* = 2~2.3 μm [33,34,35]. In the model, we assume that the ellipsoid is a truncated cone, a structure that reduces the coupling loss between the myoid and the outer segment [6]. The diameter *d_e_*_1_ of the larger section of the truncated cone is equal to the diameter of the myoid, and the smaller *d_e_*_2_ is the same as the outer segment. In Rsoft, we build four cone models with different lengths of *L*_1_~*L*_4_. Each part of *L*_1_~*L*_4_ has the same diameter and different lengths, where *d_o_* = 1.6 μm, *d_m_* = *d_e_*_1_ = 2 μm, *d_os_* = *d_e_*_2_ = 1 μm; the sum of the lengths of the four models (*L_n_ = l_o_ + l_m_ + l_e_ + l_os_*, unit: μm) is: *L*_1_ = 80 + 11 + 14 + 14, *L*_2_ = 90 + 14 + 16 + 16, *L*_3_ = 100 + 11 + 14 + 14, *L*_4_ = 100 + 16 + 20 + 20 μm. The parameters of the seven cones in the four models are the same, and the surrounding six cones are arranged in a hexagon around the central cone [10,36]. We set the distance between cones *δ_d_* = 2.1 μm. The parameters of the refractive index of each part of the model are set to *n_o_* = 1.36, *n_m_* = 1.36, *n_e_* = 1.39, *n_os_* = 1.385, and the extracellular medium is set as *n_ex_* = 1.3476 (ref. [7,10,27,28]).

### 2.4. Splitting Ratio

Monitors are set at the output of the outer segments of the seven cones, and the measured optical power is recorded. For the study of the cone model, the physical quantity of the splitting ratio is used for analysis, and the calculation formula is:

(1) $$P_{n\_\Phi_{k}}=\frac{P_{\Phi_{k}}}{\sum_{i=0}^{6}P_{\Phi_{i}}}(k=0,1,\ldots ,6)$$

where $P_{n\_\Phi_{k}}$ is the splitting ratio of the *k*-th cone model and $P_{\Phi_{k}}$ is the measured optical power of the outer segment of the *k*-th cone. The ratio of the optical power of a single cone to the total optical power of the seven cones is the splitting ratio. In the simulation, light is incident on the outer fiber of the central cone, and the arrangement of the model was symmetrical, so the light splitting ratios of the six surrounding cones are the same.

## 3. Results

### 3.1. Relative Optical Power at the Output of the Outer Segment

Figure 1A displays the structure of the cone cells, which are divided into five parts. According to the structure illustrated in Figure 1A, the light propagation model of seven cone optical fiber couplers, revealed in Figure 1B, is established by using the RSoft software, and the cones are numbered (Figure 1C). The light propagation and coupling effect in the seven cones are calculated using the BPM algorithm.

**Figure 2:**
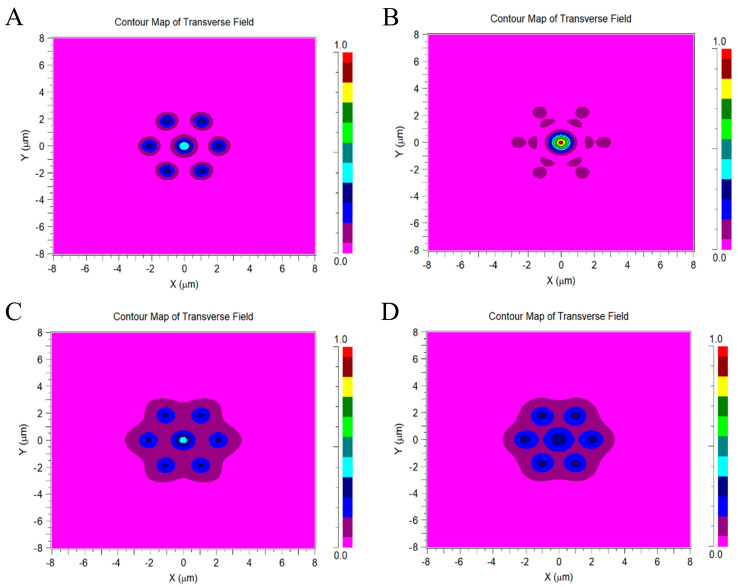
Mode field distributions at the output of the outer segment at different wavelengths. The *L*_4_ = 100 + 16 + 20 + 20 μm model is used in (**A**–**D**), and the wavelengths of the incident light used are: (**A**) λ = 430 nm, (**B**) λ = 536 nm, (**C**) λ = 690 nm, and (**D**) λ = 750 nm. The color coding on the right side indicates the magnitudes of the mode field distributions monitored at the output of the outer segment of the model.

In the simulation, the input power into the outer fiber of the central cone fiber at different wavelengths is normalized to 1, i.e., the optical power is 1 at z = 0 (μm) of the central cone. The mode field distributions at the output of the outer segment of *Φ*_0_*~Φ*_6_ are recorded by the monitor (Figure 2). The mode field distributions at the output of the outer segments are recorded at 430, 536, 690, and 750 nm. The magnitudes of the monitored mode field distributions at the output of the outer segments are judged according to the color coding of the axis shown on the right side of the figure. As shown in Figure 2A,B, when the light beams with two different wavelengths enter the central cone with the same peak amplitude, the peak mode field amplitudes at the output of the outer segment of the central cone are both higher than those of the surrounding cones. This shows that when light with a wavelength of 430 nm is incident on the central cone, most light energy is coupled into the surrounding cones at the output of the outer segment. The peak mode field amplitude in the central cone is only slightly higher than that in the surrounding cones at a wavelength of 430 nm. At the wavelength of 536 nm, most energy is retained in the central cone, while almost no energy enters the surrounding cones. As shown in Figure 2B–D, in the wavelength range of 536–750 nm, the peak mode field amplitudes of the central cone decrease gradually with increasing wavelength.

### 3.2. Relationship between Splitting Ratio and Wavelength

**Figure 3:**
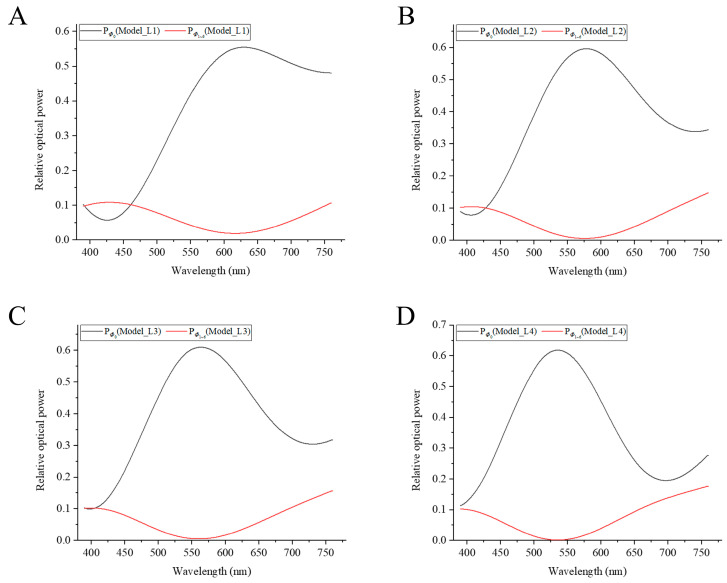
Relationship between the relative optical powers at the output of the outer segment and wavelength of the four models *L*_1_*~L*_4_. (**A**) *L*_1_ = 80 + 11 + 14 + 14 μm. (**B**) *L*_2_ = 90 + 16 + 20 + 20 μm. (**C**) *L*_3_ = 100 + 11 + 14 + 14 μm. (**D**) *L*_4_ = 100 + 16 + 20+ 20 μm. The relative optical power of the *Φ*_0_ detected at the output of the outer segment of the central cone is represented by a black curve, and the relative optical power detected at the output of the outer segment of a single surrounding cone *Φ*_1_*~Φ*_6_ is represented by a red curve.

To study the coupling effect of the four parts of the optical cone fiber couplers, we construct four models with four different length combinations of *L*_1_*~L*_4_. In the simulation, the input power into the outer fiber of the central cone fiber at different wavelengths is normalized to 1, i.e., the optical power is 1 at z = 0 (μm) of the central cone. The relative optical power at the output of the outer segment of *Φ*_0_*~Φ*_6_ are recorded by the monitor. As shown in Figure 3, the relative optical powers versus wavelength at the output of the outer segments of the four models are plotted, where black and red curves correspond to the central cone and a surrounding cone respectively. During the propagation of light, a portion of the energy escapes to the surrounding space among the seven fibers, and this results in excess losses. Therefore, the total relative optical power is different from 1.

**Figure 4:**
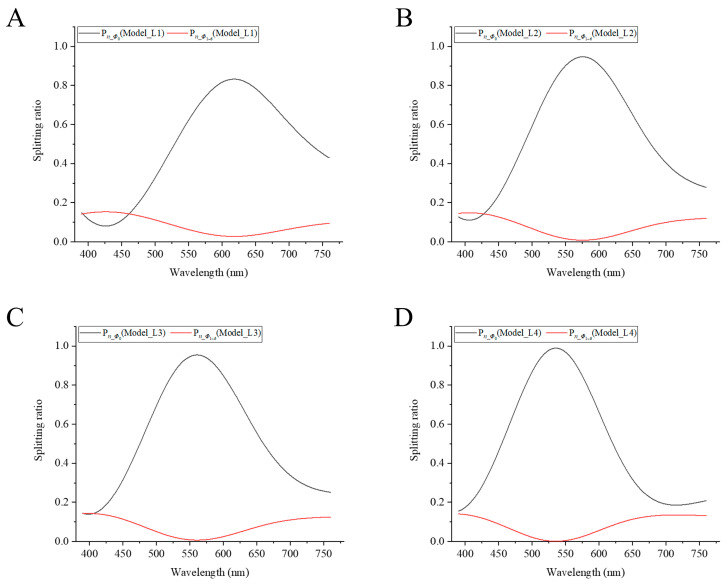
Splitting ratio curves of the central and surrounding cones vs. wavelength in four models, where (**A**) *L*_1_ = 80 + 11 + 14 + 14 μm. (**B**) *L*_2_ = 90 + 16 + 20 + 20 μm. (**C**) *L*_3_ = 100 + 11 + 14 + 14 μm. (**D**) *L*_4_ = 100 + 16 + 20 + 20 μm. The black curve represents the splitting ratio of the central cone *Φ*_0_, while the red curve represents the splitting ratio of a single surrounding cone *Φ*_1_*~Φ*_6_.

As shown in Figure 4, the total optical powers at the output of the outer segments of the seven cones at each wavelength are normalized to 1. The optical powers in the individual cones are normalized, and the couplers’ splitting ratios are calculated.

The peak wavelengths of the central cone splitting ratios are 530–630 nm in the four models (Figure 4A–D). In the four models, within a wavelength region of ±75 nm near to the peak wavelength, the splitting ratios at the output of the outer segment of the central cone are always greater than the sum of the splitting ratios of the six surrounding cones. The splitting ratios of the central cones are greater than 80% at peak wavelengths. As shown in Figure 4A, the splitting ratio of the central cone is smaller than that of the surrounding cones within a wavelength region of 390–455 nm. As shown in Figure 4B–D, the trends of the splitting ratios curves are almost the same: the splitting ratio curves are bell-shaped. In the short and long wavelength regions, the differences between the splitting ratios of the central cones and those of surrounding cones become smaller. In these two wavelength ranges, after the light enters the outer fiber of central cone, most of the energy is coupled to the surrounding cones after passing through the four layers of cone couplers. The power appears to distribute uniformly approximately at the output of the outer segments of the seven cones at some short wavelengths.

The coupling effect is related to the parameters of the model and the wavelength of the incident light. In the visible light range, there are two strong coupling regions, which are in the wavelength ranges of violet and red light, respectively. In these two strongest coupling regions, most of the energy is transferred to the surrounding cones, and only a small part is retained in the central cone. In the wavelength range of green to yellow light, most of the energy is retained in the central cone. The lengths of each part of the model also affect the coupling effect (Figure 4A,C). When the total length (of the first three parts mainly) is longer, the peak wavelength of the splitting ratio shifts to a shorter wavelength direction.

Based on our simulations, the existence of strong optical coupling among the seven cones in the central of foveal is confirmed. The optical coupling effect of cones depends not only on the wavelength of the incident light and cone’s parameters (i.e., the length, diameter, and refractive index of the various parts), but also on extracellular medium parameters (including refractive index and cones’ spacing) etc.

In the center of the foveal, there are no S cones, which are responsible for the short-wavelength light signals. The coupling effect may be one important reason for this phenomenon. When the short short-wavelength light is incident on the central cone, most of the light is coupled into the surrounding cones at the output of the outer segment or roughly evenly distributes in the incident surrounding area. If there had been S cones in the center of the foveal, the coupling effect would make the brain judge the original input location of the stimulating cone more difficult. In our two layers of the two cone fiber coupler model and three layers of the seven coupler model, we have already found this phenomenon, and we verify that a similar phenomenon happens in these four layers of the seven cone fiber coupler models again [7,10].

### 3.3. Photopic Luminous Efficiency

In the above two subsections, we analyzed the fiber coupler effect of the cones without considering the absorption of the cones. To explain the photopic luminous efficiency curve, both the fiber coupler effect and absorption of light should be considered. In this subsection, we consider the absorption of light by the cones. The classification of cones is based on the sensitivity of different opsins in the outer segment. Photopic vision is formed by the activity of only cone cells in the human eye in a bright environment, and L and M cones are the most abundant types of cones in the human eye. The spectral sensitivity data on the L cone are related to the waveguide effect and absorption coefficient of the L cone. The data on the photopic luminous efficiency of the human eye and the spectral sensitivity data of the L cone are from the literature [18,19,20].

**Figure 5:**
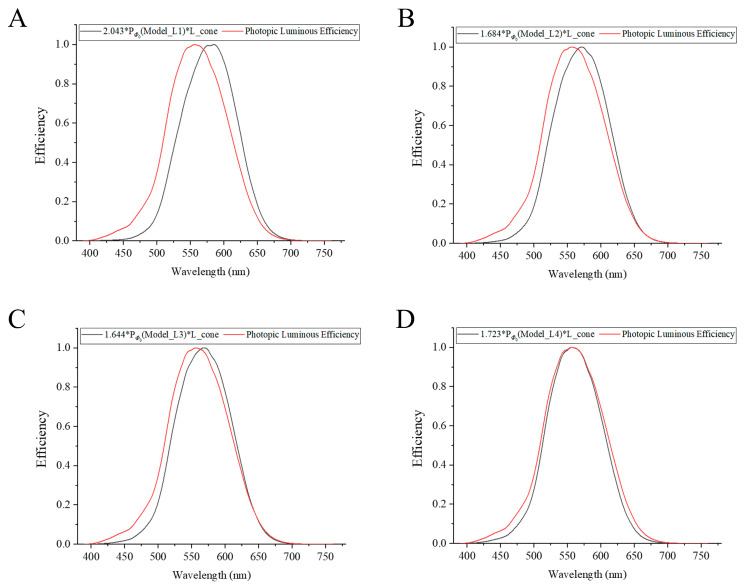
To apply γ * *P_Φ_*_0_ (Model_Li) * L_cone to fit the photopic luminous efficiency curve with i = 1–4, where, (**A**) γ = 2.043, (**B**) γ = 1.684, (**C**) γ = 1.644, and (**D**) γ = 1.723.

As shown in Figure 5, by taking into account both the fiber coupling effect of the cone photoreceptors and absorption of light, we find that the multiplication of the relative optical power of the central cone by the L cone’s spectral sensitivity is proportional to the isotopic luminous efficiency approximately. Therefore, we apply γ* *P_Φ_*_0_ (Model_Li) * L_cone to fit the photopic curve with i = 1–4, where γ is a normalization coefficient. As shown in Figure 5D, while the peak wavelength is 564 nm in the spectral sensitivity curve of the L cone, the peak wavelength of the fitted curve is shifted near to the peak wavelength 555 nm of the photopic curve. The fitted curve matches with photopic luminous efficiency well, so it shows the dominant role of the L cone in photopic luminous efficiency. Meanwhile, the L cone may contribute to the photopic luminous efficiency the most, followed by the M cone, while the S cone contributes the least [4,5,6,7,8,9,10].

## 4. Discussion

In this study, we apply the cone fiber couplers theory to explain the color pre-processing role of seven cones, and we studied the splitting ratio of the seven-cone fiber couplers made of four layers of seven cones. The splitting ratio curves reflect how the cone cells process the information carried by the different colors of light, and these show that the fiber coupling effect may play important roles in opponent color processing (yellow–blue and red–green opponent colors). There are two wavelength regions (short and long wavelength regions) with strongest coupling, where the outer segment of the central cone is no longer responsible for receiving most of the optical power. The seven-cone couplers have strong wavelength selectivity to the incident light, where the relative optical powers at the output of the outer segments are related to wavelength, cones’ parameters (including lengths, diameters, refractive index), and extracellular medium parameters (including refractive index and cones’ spacing) etc. We also studied the coupling effects by using more parameter values than shown in this paper. Our study may help to understand color blindness and weakness more as well.

By taking account of both the fiber coupling effect and absorption, we find the multiplication of the relative optical power of cone couplers by the spectral sensitivity of the L cone and a normalized coefficient that matches with the photopic luminous efficiency of the human eye well. In this study, we only consider the role of the L cone in photopic luminous efficiency but not the role of the M cone. However, the photopic luminous efficiency is mainly due to the L cones, followed by the contribution of the M cones, while the contribution of the S cones is very small [4,5,6,7,8,9,10]. Regarding human eye color perception, the formation of lightness, hue, and saturation may be mainly related to the three cones respectively. The lightness may be mainly affected by the L cones, followed by the M cones, while the S cones have the least effects; the hue may be affected by the S cones mainly; and the saturation may be affected by the M cones mainly [4,5,6,7,8,9,10]. These three cones thus work together to form the feelings of lightness, hue, and saturation in human color vision.

Through this study, we show how the optical fiber coupling effect of the cones play an important role in human vision. Our proposed bio-optical AI of the human eye is composed of four layers of fiber couplers and saturable absorbers. Among them, the outer fiber, myoid, ellipsoid, and the outer segment of the central and surrounding cones act as fiber couplers, while the outer segments of the central cones act as saturable absorbers. This bio-optical AI structure makes the human eye be a very sophisticated organ with great energy saving, heat saving and low delay [4,5,6,7,8,9,10].

In this paper, we study the coupler effect of the cones with cylindrical shape. However, the shape of cones may not be ideal cylindrical shapes in reality, we shall do further study on the influence of cone shapes on the coupling effect in future.

## 5. Conclusions

In this paper, we build the four-part cone models to explore the coupling effect of seven cone fiber couplers. Moreover, this is the first study of the coupling effect of four layers of biological couplers in animals and other biological lives. There are two wavelength regions (short and long wavelength regions) with the strongest coupling, where the outer segment of the central cone is no longer responsible for receiving most of the optical power. However, in the wavelength range of green to yellow light, most of the energy is retained in the central cone. In our four models, within a wavelength region of ±75 nm near to the peak wavelengths, located in the yellow–green region, the splitting ratios at the output of the outer segment of the central cone are always greater than the sum of the splitting ratios of the six surrounding cones. These cone couplers may play an important role in color preprocessing (e.g., partially performing opponent color processing). By taking account of both the cone fiber coupling effect and absorption of the outer segment of the L cone, we find the multiplication of the relative optical power of cone couplers, the spectral sensitivity data of the L cone, and a normalized coefficient that matches with the photopic luminous efficiency of the human eye well. This is the first attempt to use both the cone fiber coupling effect and the absorption of L cones to explain photopic luminous efficiency. The splitting ratios of the central cones are greater than 80% at peak wavelengths located in the yellow–green wavelength range, and this can help to explain why the human eye is more sensitive to green light.

Our proposed bio-optical AI on the human eye is composed of four layers of fiber couplers and saturable absorbers on the cones. The results presented here could provide new evidence for optical AI on the human eye.

## Data Availability

Not applicable.

## References

[1] Enoch J.M. (1961). Visualization of wave-guide modes in retinal receptors. Am. J. Ophthalmol..

[2] Harte R.A. (1961). Receptor elements of the human retina as semiconductors. J. Opt. Soc. Am..

[3] Liang A. Photoreceptors of Animals Are Quantum-Well Detectors. Proceedings of the Japan Society of Applied Physics 59th Autumn Meeting.

[4] Liang A., Hu L. Novel optical waveguide theory and novel electrical circuit theory of photoreceptors in the human retina. Invited talk. Proceedings of the Progress in Electromagnetics Research Symposium.

[5] Liang A., Li W., Luo X., Gu J., Liu Y., Hu L. Strong optical coupling between neighboring cones on human retina. Proceedings of the OSA Fall Vision Meeting.

[6] Liang A., Hu L., Liang Z. (2015). Mode-Field-Diameter and the Coupling Loss between Inner and Outer Segment of Photoreceptors. Opt. Photonics J..

[7] Liang A., Meng Z. (2016). The explanation of color vision based on fiber coupler theory of foveal cones. Chin. Sci. Bull..

[8] Liang A., Wang X., Meng Z. Existence of strong optical coupling among seven identical human foveal cones. Proceedings of the OSA Fall Vision Meeting.

[9] Liang A. Smart and advanced PIC on human retina and optical fibers in animal bodies. Proceedings of the SPIE.

[10] Liang A.H. Biological Optical AI and Bionic Optical AI. Plenary speech. Proceedings of the 2020 5th International Conference on Robotics and Automation Engineering (ICRAE).

[11] Ball J.M., Chen S., Li W. (2022). Mitochondria in cone photoreceptors act as microlenses to enhance photon delivery and confer directional sensitivity to light. Sci. Adv..

[12] Xin H., Li Y., Liu X., Li B. (2013). Escherichia coli-based biophotonic waveguides. Nano Lett..

[13] Xin H., Li Y., Li B. (2015). Bacteria-based branched structures for bionanophotonics. Laser Photonics Rev..

[14] Pan T., Lu D., Xin H., Li B. (2021). Biophotonic probes for bio-detection and imaging. Light Sci. Appl..

[15] Lee H.J., Ha J.H., Kim S.G., Choi H.K., Kim Z.H., Han Y.J., Kim J.I., Oh Y., Fragoso V., Shin K. (2016). Stem-piped light activates phytochrome B to trigger light responses in Arabidopsis thaliana roots. Sci. Signal..

[16] Xiaoling L. (2018). Visual Neurophysiology.

[17] Brainard D.H. (2019). Color, Pattern, and the Retinal Cone Mosaic. Curr. Opin. Behav. Sci..

[18] Stockman A. (2019). Cone fundamentals and CIE standards. Curr. Opin. Behav. Sci..

[19] Stockman A., Sharpe L.T. (2000). The spectral sensitivities of the middle- and long-wavelength-sensitive cones derived from measurements in observers of known genotype. Vis. Res..

[20] Stockman A., Sharpe L.T., Fach C. (1999). The spectral sensitivity of the human short-wavelength sensitive cones derived from thresholds and color matches. Vis. Res..

[21] Ding J., Yao Q., Jiang L. (2019). Comparisons of Scotopic/Photopic Ratios Using 2- and 10-Degree Spectral Sensitivity Curves. Appl. Sci..

[22] Snyder A.W., Hamer M. (1972). The light-capture area of a photoreceptor. Vis. Res..

[23] Gorrand J.-M., Delori F.C. (2009). A model for assessment of cone directionality. J. Mod. Optic..

[24] Snyder A.W., Pask C. (1973). The Stiles-Crawford effect—Explanation and consequences. Vis. Res..

[25] Liu Z., Kocaoglu O.P., Turner T.L., Miller D.T. (2015). Modal content of living human cone photoreceptors. Biomed. Opt. Express.

[26] Barer R. (1957). Refractometry and interferometry of living cells. J. Opt. Soc. Am..

[27] Enoch J.M., Tobey F.L. (1978). Use of the waveguide parameter V to determine the difference in the index of refraction between the rat rod outer segment and the interstitial matrix. J. Opt. Soc. Am..

[28] Spaide R.F., Curcio C.A. (2011). Anatomical correlates to the bands seen in the outer retina by optical coherence tomography: Literature review and model. Retin. J. Ret. Vit. Dis..

[29] Thylén L. (1983). The beam propagation method: An analysis of its applicability. Opt. Quant. Electron..

[30] Feit M.D., Fleck J.A. (1978). Light propagation in graded-index optical fibers. Appl. Opt..

[31] Labin A.M., Safuri S.K., Ribak E.N., Perlman I. (2014). Müller cells separate between wavelengths to improve day vision with minimal effect upon night vision. Nat. Commun..

[32] Kreysing M., Boyde L., Guck J., Chalut K.J. (2010). Physical insight into light scattering by photoreceptor cell nuclei. Opt. Lett..

[33] Sawides L., de Castro A., Burns S.A. (2017). The organization of the cone photoreceptor mosaic measured in the living human retina. Vis. Res..

[34] Litts K.M., Cooper R.F., Duncan J.L., Carroll J. (2017). Photoreceptor-Based Biomarkers in AOSLO Retinal Imaging. Investig. Ophthalmol. Vis. Sci..

[35] Foote K.G., Loumou P., Griffin S., Qin J., Ratnam K., Porco T.C., Roorda A., Duncan J.L. (2018). Relationship Between Foveal Cone Structure and Visual Acuity Measured with Adaptive Optics Scanning Laser Ophthalmoscopy in Retinal Degeneration. Investig. Ophthalmol. Vis. Sci..

[36] Burns S.A., Elsner A.E., Sapoznik K.A., Warner R.L., Gast T.J. (2019). Adaptive optics imaging of the human retina. Prog. Retin. Eye Res..

